# Pro-Inflammatory Diet Is Associated with Adiposity during Childhood and with Adipokines and Inflammatory Markers at 11 Years in Mexican Children

**DOI:** 10.3390/nu12123658

**Published:** 2020-11-27

**Authors:** Sofia Barragán-Vázquez, Ana Carolina Ariza, Ivonne Ramírez Silva, Lilia Susana Pedraza, Juan A. Rivera Dommarco, Eduardo Ortiz-Panozo, Elena Zambrano, Luis A. Reyes Castro, Nitin Shivappa, James R. Hébert, Reynaldo Martorell, Aryeh D. Stein, Albino Barraza-Villarreal, Isabelle Romieu, Laura Avila-Jiménez, Usha Ramakrishnan

**Affiliations:** 1 Center for Nutrition and Health Research, Instituto Nacional de Salud Pública, Av. Universidad 655, Col. Santa María Ahuacatitlán, Cerrada los pinos y Caminera, Cuernavaca C.P. 62100, Morelos, Mexico; sofia.barragan@insp.mx (S.B.-V.); carolina.ariza@insp.mx (A.C.A.); lilia.pedraza@espm.insp.mx (L.S.P.); jrivera@insp.mx (J.A.R.D.); 2 Center for Population Health Research, Instituto Nacional de Salud Pública, Av. Universidad 655, Col. Santa María Ahuacatitlán, Cerrada los pinos y Caminera, Cuernavaca C.P. 62100, Morelos, Mexico; eduardo.ortiz@insp.mx (E.-O.P.); abarraza@insp.mx (A.B.-V.); iromieu@gmail.com (I.R.); 3 Department of Reproductive Biology, Instituto Nacional de Ciencias Médicas y Nutrición Salvador Zubirán, Mexico City C.P. 14080, Mexico; zamgon@yahoo.com.mx (E.Z.); lafe_mat@hotmail.com (L.A.R.C.); 4 Cancer Prevention and Control Program and Department of Epidemiology and Biostatistics, University of South Carolina, 915 Greene Street, Suite 241, Columbia, SC 29208, USA; shivappa@email.sc.edu (N.S.); JHEBERT@mailbox.sc.edu (J.R.H.); 5 Hubert Department of Global Health, Rollins School of Public Health, Emory University, Atlanta, GA 30322, USA; rmart77@emory.edu (R.M.); aryeh.stein@emory.edu (A.D.S.); uramakr@emory.edu (U.R.); 6 Coordinación Auxiliar Médica de Investigación en Salud, Delegación Estatal Morelos, Instituto Mexicano del Seguro Social, Morelos C.P. 62000, Mexico; laura.avilaj@imss.gob.mx

**Keywords:** inflammation, diet, children’s dietary inflammatory index, leptin/adiponectin ratio, CRP, IL-6, Mexican children

## Abstract

There is limited evidence about the inflammatory potential of diet in children. The aim of this study was to evaluate the association between the Children’s Dietary Inflammatory Index (C-DII) from 5 to 11 years with adiposity and inflammatory biomarkers in Mexican children. We analyzed 726 children from a birth cohort study with complete dietary information and measurements to evaluate adiposity at 5, 7 and 11 y and 286 children with IL-6, hsCRP, leptin and adiponectin information at 11 y. C-DII trajectories were estimated using latent class linear mixed models. We used linear mixed models for adiposity and logistic and multinomial regression for biomarkers. In girls, each one-point increase in C-DII score was associated with greater adiposity (abdominal-circumference 0.41%, *p* = 0.03; skinfold-sum 1.76%, *p* = 0.01; and BMI Z-score 0.05, *p* = 0.01). At 11 y the C-DII was associated with greater leptin (34% ≥ 13.0 ng/mL, *p* = 0.03) and hsCRP concentrations (29% ≥ 3.00 mg/L, *p* = 0.06) and lower adiponectin/leptin ratio (75% < 2.45, *p* = 0.02). C-DII trajectory 3 in boys was associated with a 75.2% (*p* < 0.01) increase in leptin concentrations and a 37.9% decrease (*p* = 0.02) in the adiponectin/leptin ratio. This study suggests that the inflammatory potential of diet may influence adiposity in girls and the homeostasis of adipose tissue and chronic subclinical inflammation in 11-year-old children.

## 1. Introduction

Worldwide, the prevalence of childhood overweight and obesity has increased in the past decades. Excess weight gain during childhood is a risk factor for obesity in adulthood and early development of diabetes, hypertension, hyperlipidemia, and cardiovascular disorders, among other metabolic disorders [1]. Adipose tissue-derived signaling molecules such as leptin and adiponectin are fundamental for physiological systemic energy homeostasis. Several studies have shown that adiposity and body fat distribution are positively and negatively associated with leptin and adiponectin levels, respectively [2,3]. Moreover, as age increases towards puberty, adipokines and other hormones play an important role in body composition, leading to differences by gender as has been previously observed [4]. Excessive accumulation of adipose tissue and increased secretion of proinflammatory mediators including acute phase proteins (e.g., C Reactive Protein [CRP]), cytokines (e.g., Interleukin 6 [IL-6]) and some adipokines create a state of chronic, low-grade systemic inflammation that provides a substrate for disease-related processes such as insulin resistance, which is associated with metabolic syndrome and type 2 diabetes mellitus [5,6]. The adiponectin/leptin ratio has been proposed as an index of adipose tissue dysfunction, as well as a predictive biomarker for metabolic syndrome, showing a negative correlation with other biomarkers of low-grade chronic inflammation [7]. Although it has been recognized that subacute inflammation and prothrombotic states are present in childhood [8], information regarding the association between chronic low-grade inflammation in children and chronic disease development later in life is lacking.

Dietary factors have been associated with chronic inflammation previously [5,9]. Western-type energy-dense diets high in red meats, refined grains, saturated and trans-fatty acids have shown positive associations with pro-inflammatory biomarkers [5,9]. The inflammatory potential of the diet has been evaluated through a priori dietary patterns such as the Children’s Dietary Inflammatory Index (C-DII^TM^), which is a recently validated tool, based on the Dietary Inflammatory Index (DII^®^) originally published in 2014 [10], that estimates the inflammatory potential of children’s and adolescent’s diet in a continuous scale from anti-inflammatory to pro-inflammatory [11].

To our knowledge, the C-DII has been evaluated only in high-income countries, and there is no information about its association with adipokines such as leptin and adiponectin, as well as other inflammatory biomarkers in children from low and middle-income countries. Moreover, existing evidence with adiposity has been focused only on single cross-sections in time rather than evaluating diet longitudinally. Thus, the aim of this study was to evaluate the association between the inflammatory potential of the children’s diet (through C-DII) and adiposity indicators from 5 to 11 years and to examine associations with IL-6, high-sensitivity CRP (hsCRP), leptin and adiponectin concentrations at 11 years in Mexican children.

## 2. Materials and Methods 

### 2.1. Study Design and Participants

This study included children of women who participated in the POSGRAD study (Prenatal Omega-3 fatty acid supplementation and child growth and development). A detailed description of the study methodology has been published elsewhere [12]. The original study began in February 2004 with 1094 pregnant women who were recruited at the Mexican Social Security Institute (IMSS) located in Cuernavaca, Mexico. Eligibility criteria for the original study included being between 18 to 35 years old, in gestational weeks 18 to 22, planning to deliver at the IMSS General Hospital in Cuernavaca, exclusively or predominantly breastfeed for at least 3 months, and living in the area for at least 2 years after delivery. Exclusion criteria included high-risk pregnancy, lipid metabolism or absorption disorders, regular intake of fish oil or docosahexaenoic acid (DHA) supplements or chronic use of certain medications (e.g., epilepsy). Eligible, consenting women were randomly assigned to receive a daily dose of either 400mg of DHA or placebo from mid-pregnancy to delivery; thenceforth, children have been followed prospectively as a birth cohort [12]. Exclusion criteria for children included congenital disorders. During follow up visits, a questionnaire was applied to evaluate children´s health status, and the diagnosis of chronic disease was used as additional exclusion criteria. Moreover, at the 11 years visit, the presence of acute diseases (such as viral infections and common cold) were added as exclusion criteria for blood samples.

**Figure 1:**
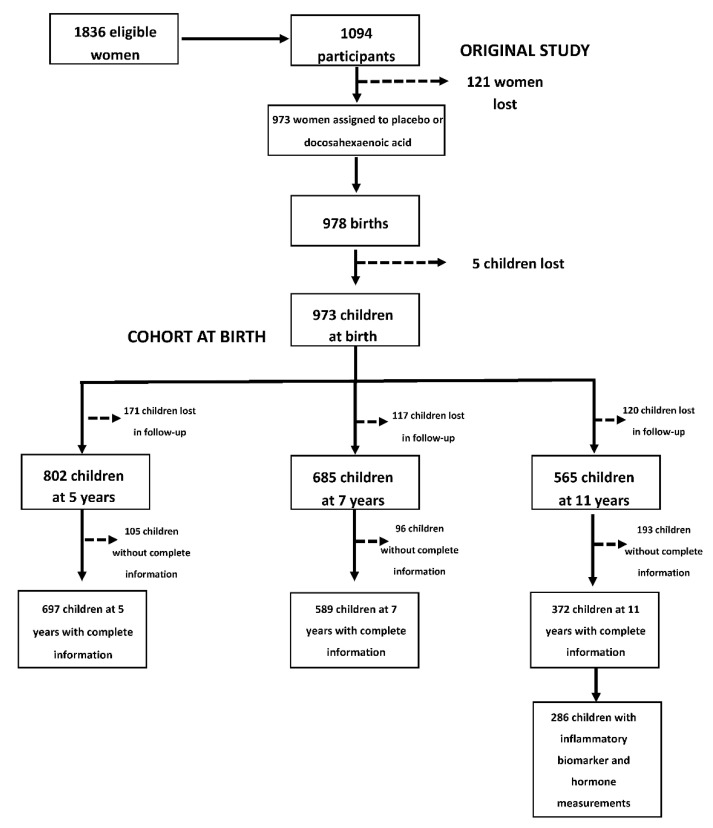
Prenatal Omega-3 fatty acid supplementation and child growth and development (POSGRAD) birth cohort study sample selection.

This study considered 726 children with complete information on dietary indicators (from 24-h dietary recalls), anthropometric measurements and other characteristics of interest (such as type of breastfeeding, among others) in at least one time point from 5–11y (5y *n* = 697; 7y *n* = 589 and 11y *n* = 372). On average, children had information on 2.3 time points. A total of 372 children had complete information on dietary, sociodemographic characteristics of interest and anthropometric indicators at 11y, and 286 had serum concentrations of hsCRP, IL-6, adiponectin, and leptin at 11y (Figure 1).

The information and procedures in this study were obtained in accordance with the guidelines of the Declaration of Helsinki. The study was approved by the Institutional Review Board at Emory University and the Research and the Ethics Committee of the National Institute of Public Health of Mexico.

### 2.2. Study Variables

#### 2.2.1. Exposure Variable

The inflammatory potential of the children’s diet was evaluated though the Children’s Dietary Inflammatory Index (C-DII). The C-DII methodology has been described elsewhere [11]. In summary, C-DII is based on the Dietary Inflammatory Index (DII^®^) [10], which is a population-based index designed to estimate the effect that the intake of 45 food parameters have on IL-6, IL-1β, IL-4, IL-10, TNF-α and hsCRP concentrations. Intake of these food parameters is compared to a world reference dietary database as Z-scores that are then converted into centered proportions and multiplied by the overall food parameter-specific inflammatory effect score. This world reference dietary database was constructed to represent the diversity of children’s diets from 16 different countries, representing 6 continents [11]. Finally, the estimated food parameter scores are added to obtain continuous scores that fall within the theoretical range of −8.87 (highly anti-inflammatory diet) to +7.98 (highly pro-inflammatory diet) but typically ranges from about −6 to about +6. The C-DII is an energy-adjusted index that is derived by comparing to a children’s world standard dietary database and is based in 25 food parameters instead of 45. The validation of the C-DII was carried out with 3300 children aged 6–14 years from the National Health and Nutrition Examination Survey (NHANES) with hsCRP measurements, where a significant positive association was observed [11]. For this study, we derived C-DII scores and assigned C-DII tertiles for 5, 7 and 11y as low, neutral and high dietary inflammatory potential, respectively.

Dietary information for the C-DII was collected through the Automated Software of the multiple-pass 24-h dietary recall (MP-24HR) version 1.0 (NIPH, 2012, Mexico) at 5, 7 and 11y. Interviews were administered by trained personnel to the child’s primary caregiver (in presence of the child) on randomly selected weekdays, including weekends. The methodology used was the five-step multiple-pass method developed by the United States Department of Agriculture (USDA) [13], which is used to avoid under-reporting of dietary intake. A group of researchers from the National Institute of Public Health (NIPH) adapted the multiple-pass method to the Mexican context for its use in the Mexican population [14]. The dietary information collected was processed according to the methodology of ENSANUT-2016 [15], and the nutrient and energy estimations were obtained with the 2012 [16] and 2016 [17] Mexican Food Database (BAM in Spanish): compilation of the frequently consumed foods in the country in its 18.1.2 and 18.1.1 versions, which are indirect compilations from several food composition databases constructed by the Center for Nutrition and Health Research of the NIPH. This was done to account for industrialized product reformulations due to taxation on sugar sweetened beverages and energy-dense foods in 2013. Estimates obtained included individual dietary values for energy, carbohydrates, protein, fiber, total fat, saturated fat, monounsaturated fatty acids (MUFA), polyunsaturated fatty acids (PUFA), vitamin A, thiamine, riboflavin, niacin, vitamin B6, folic acid, vitamin B12, vitamin D, vitamin C, vitamin E, ¦Â-carotene, cholesterol, alcohol, iron, magnesium, selenium and zinc.

#### 2.2.2. Outcome Variables

Adiposity. Body Mass Index (BMI) Z scores, abdominal circumference and skinfold sum from 5, 7 and 11y were the indicators considered to evaluate adiposity in the longitudinal models because fat mass was only available at 11y. 

To obtain the BMI Z scores, we used the World Health Organization’s (WHO) “AnthroPlus” Macro for Stata, which is based on the WHO 2007 5–19 years growth references [18]. Weight, height, abdominal circumference and skinfolds (tricipital, bicipital, subscapular and suprailiac) were measured at 5, 7 and 11 years by standardized personnel using international procedures [19,20]. Children were weighed wearing light clothing with a portable Tanita electronic pediatric scale (model 1582) with a precision of 100 g, which was calibrated daily with a known reference weight. Height was measured using a stadiometer with a precision of 0.1 cm. Abdominal circumference was measured with a fiberglass tape with a precision of 0.1 cm. All measurements were performed in duplicate, and the average value was calculated. Skinfolds were measured in triplicate on the left side of the body, when the child was right-handed, and on the right side, when the child was left-handed, with a Lange skinfold caliper to the nearest 1.0 mm, while applying a constant pressure of 10 g/mm^2^; the mean value was used in the analyses.

### 2.3. Inflammatory Biomarkers and Adipokines at 11 Years

Serum was obtained after a 12 h fasting period, divided into aliquots, frozen in liquid nitrogen and kept at −70 °C until further assays.

High sensitivity C reactive protein (hsCRP) determination was made in serum by using a latex immunoanalysis methodology in which antigen-antibody reaction is absorbed by latex particles and detected at 572 nm. Absorbance was measured with an ARCHITECT C8000 equipment (ABBOTT, Illinois, USA-TOSHIBA, Tokio, Japan).

Interleukin (IL-6) determination was made using a commercial kit for human IL6 (R&D Systems, Minneapolis, MN, USA) following manufacturer’s instructions. Standard curve and sample measurements were performed at 450 nm. Absorbance measurement (450 nm) was carried out.

Serum leptin concentrations were determined using a specific commercial kit for human leptin (Merck-Millipore, Burlington, MA, USA, EZHL-80SK). Standard curve and sample measurements were obtained at 450 nm.

Adiponectin concentrations were analyzed using a commercial kit for human adiponectin (Merck-Millipore, EZHADP-61K) following the manufacturer´s instructions with some modifications: a standard curve was prepared as indicated and serum samples were used undiluted. After washing, standards or samples were incubated with 20 µL of antibody at room temperature for 2 h. After 3 times of washing, the enzyme solution was added and incubated for 30 min. A 100 µL substrate solution was added after a 5-time washing and incubated for 15 min. The reaction was stopped, and absorbance was measured at 450 nm–590 nm.

All biomarker concentrations were obtained from a single measurement per sample. hsCRP has a lower detection limit of 0.19 mg/L and had an intra-assay variation of 4.20%. Intra and inter-assays variation and sensitivity for leptin, adiponectin and IL6 were 8.2% and 8.5% with 0.5 ng/mL; 8.9% and 9.4% with 1.5 ng/mL; 7.56% and 7.95% with 0.7 pg/mL, respectively.

Adiponectin/leptin ratio was calculated to measure adipose tissue dysfunction and consider the relationship between serum concentrations of adiponectin and leptin.

### 2.4. Covariates

Covariates used in this analysis were child’s age (only in the case of linear mixed models), child’s sex, child’s birth weight, household socioeconomic level close to the time of birth (socioeconomic index, constructed with information related to housing, sanitation and possession of household goods at recruitment of the mothers), body mass index of the mother at 18–22 weeks of pregnancy, maternal education (in years), DHA treatment at pregnancy (randomization process gave the original design of the study), type of breastfeeding at 3 months of age and body fat mass at 11 years (only in the case of biomarker models); this indicator was evaluated using Bod Pod air displacement plethysmography (Bod Pod Express, COSMED, CA, USA) [21,22,23].

### 2.5. Statistical Analysis

We calculated frequencies and percentages for categorical variables, means and standard deviations for normally distributed continuous variables, as well as the 50th, 25th and 75th percentiles for continuous variables without normal distributions across the different time points (5, 7 and 11 years). We estimated 50th and 25th–75th percentile values of inflammatory biomarkers and adipokine concentrations at 11 years across BMI categories and used Dunn’s test. Median differences of inflammatory biomarkers and adipokine concentrations by fat mass were calculated with quantile regressions.

We estimated the mean macronutrient contributions to dietary energy and relevant nutrients across C-DII tertiles by age (at 5, 7 and 11 years) to describe the dietary characteristics (energy, macro and micronutrients) of C-DII in Mexican children. To estimate differences between tertiles, we used one-way ANOVA.

We used linear mixed models to estimate the association of C-DII score with adiposity indicators (BMI Z score, skinfold sum and abdominal circumference) from 5–11 years. The sample size for these models was based on 726 children with a total of 1658 observations. Skinfold sum and abdominal circumference models were log-transformed to improve the model’s goodness of fit. We evaluated the potential modifying effect of sex and age in the association of C-DII score with adiposity and found a statistically significant interaction for sex. All models were adjusted for age, breastfeeding type at 3 months, child’s birth weight, socioeconomic level, maternal BMI, mother’s education, and supplementation with DHA during pregnancy. Finally, we used the naïve method to obtain the percentage of change in log-transformed models [24].

For associations between C-DII and biomarkers, we analyzed the association between (1) C-DII score at 11 years and biomarker outcomes at 11y and (2) longitudinal C-DII exposure from 5–11y with the 11y outcomes of biomarkers.

First, we derived C-DII trajectories from 5–11y with latent class linear mixed models for participants with complete information using R Studio Team (2019) (RStudio Team (2019) (RStudio: Integrated Development for R. RStudio, Inc., Boston, MA, USA, URL http://www.rstudio.com/) Models with linear, quadratic and cubic functions of time were explored. In each of them, 1 to 7 latent classes were specified without the inclusion of covariates and stratification by sex. All models were estimated using maximum likelihood. We used Bayesian Information Criterion, Akaike’s Information Criterion, entropy values and a minimum of 10% of participants in each latent class to assess goodness of fit. We obtained 2 latent classes for girls and 3 latent classes for boys, both with a quadratic function of time. The mean of posterior probabilities of classification in each class were all above 0.70. The derived latent classes were used in linear regression models with each biomarker as dependent variables. Biomarkers were log-transformed to improve the model’s goodness of fit. Results were expressed as percentage of change using the naïve method [24].

For cross-sectional associations of C-DII with biomarkers at 11 years, we used logistic regression for hsCRP, and multinomial logistic regression for IL-6, leptin, adiponectin and adiponectin/leptin ratio. The hsCRP outcome was defined as having serum concentrations ≥ 3 mg/L, based on previously established risk thresholds [25,26]. Since adiponectin, leptin, adiponectin/leptin ratio and IL-6 showed trimodal and multimodal distributions, we used finite mixture models to identify the optimal cut off point for each biomarker distribution, establishing categories as follows: (1) IL-6 reference category were those with concentrations < 0.55 pg/mL, neutral category between 0.55 and <6.23 pg/mL and risk category ≥ 6.23 pg/mL. (2) Leptin reference category were those with concentrations < 1.88 ng/mL; second category were those between 1.88 and <5.68 ng/mL; third category were those between 5.68 and <13.00 ng/mL, and the risk category were concentrations ≥13.00 ng/mL. (3) Adiponectin reference category included those with concentrations ≥ 19.63 ng/mL, neutral category included those between <19.63 and 13.76 ng/mL and risk category included <13.76 ng/mL. (4) Adiponectin/leptin ratio reference category included those ≥ 23.43; neutral category included those between <23.43 and 2.45, and risk category included <2.45. We used Bonferroni’s adjustment for multiple comparisons as appropriate. *p*-values < 0.05 were considered statistically significant. All the statistical analyses were performed using Stata V.14 (StataCorp. 2015. Stata Statistical Software: Release 14. College Station, TX, USA: StataCorp LP.).

## 3. Results

We analyzed the data of 726 children from 5 to 11 years with complete information on C-DII, covariates of interest and outcomes for adiposity and 286 children with complete information for biomarkers at 11 years, who represented the 74.6% and 29.4% of the total sample of children studied in the cohort, respectively. Comparing to those excluded, the analyzed sample for adiposity was only significantly different in terms of the prevalence of smoking during pregnancy (*p* = 0.02). For the biomarker sample at 11 years, there were significant differences regarding maternal socioeconomic level (*p* = 0.04) (Table S1).

Our study sample included 697 children followed up to 5y, 589 children followed up to 7 years, and 372 children followed up to 11 years, of which approximately 52% were male. Exclusive or predominant breastfeeding at 3 months of age was prevalent in roughly 25% of the sample. The socioeconomic level of children was evenly distributed across high, medium, and low at ages 5 and 7 but was predominantly high (40%) at age 11. Most mothers had an educational level of high school or higher across all ages, while their BMI was mostly high (around 57%) at the 5, 7 and 11 years’ time points. While most children had normal weight at all ages, 12.3%, 16% and 24.5% of children had overweight, and 6.2%, 13.4% and 18.5% of children had obesity at ages 5, 7 and 11 years, respectively. The mean abdominal circumference (cm) and skinfold sum (mm) increased steadily with age. FM was only available at 11 years and had a mean value of 13.9 Kg ± 6.9 Kg. Regarding the inflammatory potential of the children’s diets, the mean C-DII score was 0.6 at 5 years, 0.6 at 7 years and 0.9 at 11 years, reflecting a more pro-inflammatory diet when approaching adolescence. Moreover, biomarker and adipokine concentrations were available only at 11 years, with a median value of 0.78 mg/L, 0.50 pg/mL, 14.7 and 6.1 ng/mL for hsCRP, IL-6, adiponectin and leptin, respectively. Adiponectin/leptin ratio had a median value of 2.5 (Table S2).

### 3.1. Energy, Fiber and Nutrients from Diet According to Dietary Inflammatory Index Tertiles

The contribution of fiber according to C-DII score was significantly different among tertiles of the index at 5, 7 and 11 years. The low and neutral inflammatory potential of diet categories (C-DII tertiles 1 and 2, respectively) had higher energy contributions from fiber compared to the high inflammatory potential category (C-DII tertile 3) from 5 to 11 years. Differences in total fat contribution were also significant, with the low and neutral categories having a lower fat contribution to energy than the high inflammatory potential category at 5 and 7 years. However, at 11 years the contribution of fat to energy was only significantly different between those in the low versus the high inflammatory potential category. A significantly higher energy contribution of protein in the low inflammatory potential compared to those in the high inflammatory potential category was also observed across ages. Finally, those with low inflammatory potential had a significantly higher energy contribution of carbohydrates at 5 and 7 years (Figure S1). Additionally, out of the 25 food parameters, fiber, saturated fatty acids, vitamin A, β-carotene, vitamin C, vitamin E, thiamin, riboflavin, niacin, B6, folate, iron and magnesium were significantly different between C-DII tertiles at 5, 7 and 11 years (Table S3).

### 3.2. C-DII Trajectories (5–11 Years) by Sex

The 438 boys with complete information from 5-11 years were grouped into 3 trajectories: 18.5% in trajectory 1, 14.4% in trajectory 2 and 67.12% in trajectory 3. Trajectory 1 was characterized by starting in a relatively lower C-DII score at 5 years (mean C-DII ± S.D: −0.58 ± 1.39) followed by a decline at 7 years (−1.42 ± 0.88) and an increase at 11 years (0.53 ± 0.90). Trajectory 2 showed an increase in the proinflammatory potential of diet from 5 to 7 years, followed by a decrease at 11 years (mean C-DII ± S.D: 5 years 0.08 ± 1.56; 7 years 0.90 ± 1.01; 11 years −0.90 ± 1.04). Lastly, trajectory 3 was considered as the risk category since it was the most proinflammatory at 5, 7 and 11 years (Figure S2a) and showed an increase in the inflammatory potential of diet from 5 to 11 years (mean C-DII ± S.D: 5 years 1.16 ± 1.26; 7 years 1.06 ± 1.09; 11 years 1.66 ± 0.92). The 379 girls with complete information were grouped into 2 trajectories: 17.7% into trajectory 1 and 82.32% into trajectory 2. We considered trajectory 2 as the risk category because it showed a more pro-inflammatory diet than trajectory 1 in the different time points, with an increase from 5 to7 years (mean C-DII ± S.D: 0.54 ± 1.37 at 5 years; 1.22 ± 1.02 at 7) and a slight decrease at 11 years (0.88 ± 1.31). Meanwhile, trajectory 1 showed a more anti-inflammatory potential of diet at 5 and 7 years (mean C-DII ± S.D: −0.03 ± 1.46 at 5 years; −1.56 ± 0.73 at 7 years), followed by an increase in the inflammatory potential of diet at 11 years (0.95 ± 1.46; Figure S2b).

### 3.3. C-DII and Adiposity from 5 to 11 Years

Significant positive associations were identified between a higher C-DII score and greater adiposity evaluated through skinfold sum, BMI Z score and abdominal circumference in girls. For every one-unit increase in the C-DII score, skinfold sum increased in 1.76% (*p* = 0.01), abdominal circumference increased in 0.41% (*p* = 0.03), and BMI Z score increased in 0.048 units (*p* = 0.01). No significant associations were identified for boys. (Table 1).

### 3.4. C-DII (11y), Inflammatory Biomarkers and Adipokines at 11 Years

Regarding the inflammatory biomarkers and adipokine measurements and adiposity at 11 years, a statistically significant increase across BMI categories was observed for hsCRP (*p* < 0.001) and leptin concentrations (*p* = 0.02). No significant differences were observed in IL-6, adiponectin and the adiponectin/leptin ratio across the BMI categories. Fat mass was positively and significantly associated with a 0.10 mg/L increase in the median concentration of hsCRP (*p* < 0.001) and in 0.19 ng/mL of the median leptin concentrations (*p* = 0.01). No significant differences were observed with IL-6, adiponectin or adiponectin/leptin ratio (Table 2).

As for C-DII and inflammatory biomarker and adipokine concentrations, a significant association was observed between a higher C-DII score and higher leptin concentrations (*p* < 0.05) and lower adiponectin/leptin ration (*p* < 0.05). For hsCRP, a positive and marginal association was found with a higher C-DII (*p* = 0.06). No significant associations were found between C-DII and IL-6. (Table 3).

### 3.5. C-DII Trajectories (5–11 Years), Inflammatory Biomarkers and Adipokines at 11 Years

Finally, for the association between the longitudinal exposure of C-DII trajectories and inflammatory biomarkers as well as adipokine concentrations at 11 years, we observed a significant increase in leptin concentrations of 75.24% (*p* < 0.01) and a significant decrease in the adiponectin/leptin ratio of −37.89% (*p* = 0.02) in boys classified in trajectory 3 compared to trajectory 1, considered as the risk category (Table 4). No statistically significant associations were found for IL-6, adiponectin or hs-CRP concentrations.

## 4. Discussion

Our findings show that girls with a pro-inflammatory diet from 5 to 11 years had greater adiposity and that a more pro-inflammatory diet in girls and boys was associated with higher leptin and hsCRP concentrations and lower adiponectin/leptin ratio at 11 years, independently of adiposity. Furthermore, we showed that the exposure to a more pro-inflammatory diet in boys from 5–11 years is associated with an increase in leptin concentrations and a decrease in the adiponectin/leptin ratio at 11 years.

Experimental and epidemiologic studies have documented the role of diet in the regulation of chronic inflammation [27]. Our results are similar to those of Bawaked et al. [28] and Correa-Rodriguez et al. [29], who documented that in Spanish children and adolescents, those who classified in the higher DII quintiles or quartiles had higher total fat, MUFA and saturated fat intake, but a lower total protein, fiber, vitamin and mineral intake. However, our results differed for energy, carbohydrate and PUFA intakes, i.e., we did not identify significant differences across tertiles of C-DII. These findings show that within the Mexican diet, a higher intake of total fat and saturated fat and a lower consumption of fiber along with β-carotene, vitamin C, vitamin E and magnesium (known for having anti-inflammatory and antioxidant properties) [30] during extended periods in childhood could contribute to detrimental health outcomes, including a pro-inflammatory state.

In Mexico, between 1999 and 2016, there has been a significant increase in the combined prevalence of overweight and obesity in children from 5–11 years. The combined prevalence increased from 25.5% to 32.8% and from 28.2% to 33.7% in girls and boys, respectively [31,32,33]. This study reflects the prevailing Mexican nutritional situation in that the studied population showed increasing prevalence of overweight and obesity as age increased. Likewise, our results about the association between pro-inflammatory diet and adiposity were similar to those documented by Aslani et al. [34] and Ramallal et al. [35], who found that children in the highest DII quartile had higher BMI z-score (*p* < 0.05) and higher hazard ration for obesity 1.32 (95% confidence interval 1.08–1.60) compared with children with lowest DII quartile, respectively. Moreover, Navarro et al. [36] found a significant association between a pro-inflammatory C-DII and childhood obesity at 5 years (OR *=* 1.09, *p* = 0.02) and overweight/obesity at 5 and 9 years (OR = 1.06, *p* = 0.01 and OR = 1.12, *p* = 0.01, respectively).

It is important to mention that for adiposity, adiponectin, leptin and adiponectin/leptin ratio models, we performed analysis adjusting by breast button development for the girls (did not consider menarche period because only few girls had had the menarche period at 11y) and testosterone concentrations for boys for sexual maturity considerations (Tables S4 and S5). However, the original results did not change significantly, and since inclusion of these variables compromised the sample size (reduction in 415 subjects for adiposity models and 79 for biomarker models), we decided to keep the original models without sexual maturation indicators.

Our results in comparison with other studies differ in the fact that we only found statistically significant associations with adiposity in girls. This could be due to differences in adipose tissue distribution between boys and girls, as well as to hormonal mechanisms, since adjusting by breast button development and testosterone concentrations increased the significance of the interaction term between C-DII and sex and increasing the effect size in girls (Table S4). Some epidemiological studies have found sex related differences but do not show a consistent pattern since some of the studies find associations in boys whereas others do in girls [37,38]. It is also possible that boys have a different response to a proinflammatory diet at a similar age since boys start sexual development later than girls and that adiposity distribution is different, being the third adipose rebound first in girls (10 years) than in boys (11–12 years) [39,40]. Other factors such as the effects of hormones and fetal and early-life exposures could determine later gender dependent responses as suggested by animal models and some epidemiological studies [3,41,42,43].

Obesity is associated with an altered secretion of cytokines and adipokines, which can increase metabolic and cardiovascular risk. Leptin contributes to a pro-oxidative state resulting in oxidative stress, inflammation and vascular dysfunction whereas adiponectin increases insulin sensitivity and reduces inflammation in various cell types with a general benefit to metabolic and cardiovascular health [44,45]. Thus, the adiponectin to leptin ratio may be a useful means for estimating obesity-associated cardiometabolic risk [7]. Evidence shows that these biomarkers could predict the onset of metabolic and cardiovascular diseases and, for elevated hsCRP levels, an increase in risk of all-cause and cardiovascular mortality in the general population [46]. In this study, hsCRP was significantly higher in children with overweight or obesity whereas IL6 levels showed no differences between groups. Our results regarding hsCRP agree with several previous studies in which it has been associated in children with overweight and obesity in different populations [47,48,49]. In this study, leptin levels were significantly increased in children with overweight and obesity, as has consistently been observed in previous studies [50,51,52]. In contrast, adiponectin was not modified in our population. The reasons for this discrepancies are still unclear; however, it is possible that adiponectin production responds to adaptation mechanisms (especially in overweight children or those with mild obesity) as observed in Murdolo et al.’s [50] study, where the total adiponectin levels of children with obesity were not different from the normal-weight group and were even higher in overweight boys. Another possibility that needs to be explored is that adiponectin could be more influenced than leptin by other metabolic factors in which inter-individual variability in adipose tissue composition, distribution, physiology and sex related hormones, especially within the development of the prepubertal period, play an important role [50,53]. However, we analyzed our results adjusting by sexual maturation, finding no significant changes in the adiponectin, leptin and adiponectin/leptin ratio models (Table S5).

Regarding the association between the C-DII in different stages during childhood and inflammatory markers at 11 years, we observed a positive and marginally significant association between C-DII and hsCRP concentrations above 3 mg/L. Moreover, we identified that boys in trajectory 3, characterized by a more pro-inflammatory potential of diet from 5 to 11 years, had a significant increase of 75.24% in leptin concentrations and a decrease of 37.89% in the adiponectin/leptin ratio at 11 years. Results in girls, though not statistically significant, were in the opposite direction than what was observed in boys. This was probably due to the increase in the proinflammatory potential of diet in C-DII trajectory 1 at 11 years (increase in 2.5 units from 7 years to 11 years), while trajectory 2 had a decrease in the proinflammatory potential of 0.34 units from 7 to 11 years. Our results are similar to those observed by Shivappa et al. [54], in which the higher DII scores were associated with values of hsCRP > 3 mg/L in the SEASONS Study cohort. However, only adults were included in that study. In addition, the NHANES study conducted by Khan et al. [11] for the validation of the C-DII, showed that children with the most pro-inflammatory diets had a CRP value 0.097 mg/dL higher in contrast with children in the lowest C-DII quartile. For our population, we considered our findings physiologically relevant since hsCRP is considered a reliable inflammatory biomarker that even with small changes can predict the inflammatory state in several pathologies [55]. However, more studies are needed to confirm these observations.

In this study, we also evaluated for the first time in children the role of a pro-inflammatory diet with leptin levels. Interestingly, C-DII at 11 years was positively associated with leptin levels, and this relationship was not only significant but consistently higher in all categories for leptin that were evaluated. Previous observations in adult population have also shown that pro-inflammatory diets measured by tertiles of the DII score are associated with higher leptin levels.

For example, Luglio et al. [56] observed a statistically significant association (β = 0.096, *p* = 0.02) between DII and plasma leptin concentrations in Indonesian adults. Moreover, for the first time, we evaluated the association between the C-DII and the adiponectin/leptin ratio index, which has been suggested to be a more accurate predictor of adipose tissue dysfunction than adipokines alone. As the adiponectin/leptin ratio decreased, a positive and significant association with C-DII at 11 years was observed. In this study, adiponectin alone and IL6 were not associated with C-DII. The lack of association between the C-DII and IL-6 observed in this study may be due to the variability in IL-6 concentrations in our population, less sensitivity of this biomarker or due to ethnicity [57]. Results for this cytokine remain controversial in children; some studies in non-Hispanic children have also reported no associations [58] while others have reported a significant positive association [59]. Moreover, a recent study in Mexican adults found, over a period of 13 years, a positive association between the highest quartile of DII and the incidence of metabolic syndrome (HRQ4vsQ1 = 1.99; 95% CI: 1.03, 3.85; *P*-trend = 0.04), particularly associated with hypertriglyceridemia, hypertension and abdominal obesity [60]. Even though in that study cytokines and adipokines were not assessed, it supports the utility and importance of earlier evaluations in childhood to prevent metabolic and cardiovascular diseases.

Overall, our results suggest that diet plays a key role in the adverse inflammatory outcomes associated with obesity comorbidities, which are present since childhood.

Some limitations of this study should be considered. During the conduction of the cohort, 25.4% of children were excluded from these longitudinal analyzes due to being lost to follow up or for missing information from 5 to 11 years. We lost a considerable number of subjects to follow-up from the original cohort, and therefore, we have many missing values. However, the attrition analysis showed that the losses seemed to be largely random. Moreover, biomarkers were measured once per sample, but sample processing and measurements were carefully handled to avoid further variations. In addition, we note that results derive from a secondary analysis. However, we consider that the cohort design and precision in selected measurements were adequate to answer our research questions. Nonetheless, it is worth noting that estimations of C-DII at 5 years may be affected by the fact that the World Reference Dietary Database was constructed with information from children aged 6–14 years. Furthermore, we did not consider dietary supplements in C-DII calculations, which could be under estimating micronutrient intake, particularly omega 3 fatty acids, vitamin C, vitamin D and vitamin A. Moreover, our covariates did not include physical activity both in the longitudinal and cross-sectional models. However, we performed a sensitivity analysis adjusting for time in front of a screen as a proxy for physical activity and METs at the 11 years follow-up and found no significant effects in the estimates of these models (data not shown).

Finally, the lack of significance in some of the biomarker models could be due to insufficient population size, as well as to the high variability in IL-6 concentrations in our population.

Strengths of this study include that it is one of the first studies carried out in Mexican children that evaluated the inflammatory potential of diet, associated with leptin, adiponectin and inflammatory markers and also that we evaluated the longitudinal exposure of C-DII in adiposity and biomarker indicators. Furthermore, the information analyzed derived from a birth cohort, which allows adjusting for important confounders from early stages in life. Moreover, dietary information was collected through the multiple-pass 24-h dietary recall method, applied with the primary caregiver present, which is the recommended instrument for C-DII and DII calculations and has proven to diminish under-reporting of dietary intake. Finally, body fat mass at 11 years was measured using Bod Pod, which is a gold standard method for body composition both in adults and children.

In conclusion, our results showed that high C-DII during childhood is associated with greater adiposity. Likewise, a high C-DII is associated with higher leptin and hsCRP concentrations and lower adiponectin/leptin ratio in Mexican children at 11 years of age, independently of fat mass. Our results suggest that the inflammatory potential of diet may influence adiposity in girls and the homeostasis of adipose tissue inflammation and systemic inflammation in 11-year-old children. The evaluation of the effect of a pro-inflammatory diet could greatly contribute both to understanding of the relationship between inflammation, adiposity and its comorbidities and to designing nutritional interventions at key stages during childhood, especially in countries where overweight and obesity represent a public health challenge.

## Figures and Tables

**Table 1:** Association between C-DII and adiposity indicators from 5–11 years ^1^.

Children’s Dietary Inflammatory Index	BMI Z Score	Log-Abdominal Circumference	Log-Skinfold Sum
**C-DII boys**			
β (95% CI)	0.016 (−0.019–0.050)	0.000 (−0.003–0.003)	0.004 (−0.008–0.016)
% of change	-	0.01 (−0.33–0.35)	0.42 (−0.79–1.65)
*p* value	0.37	0.96	0.49
**C-DII girls**			
β (95% CI)	0.048 (0.011–0.085)	0.004 (0.00–0.008)	0.017 (0.004–0.030)
% of change	-	0.41 (0.05–0.78)	1.76 (0.46–3.08)
*p* value	0.01	0.03	0.01
***p* for C-DII-sex interaction**	0.21	0.11	0.14

BMI: Body Mass Index; CI: Confidence Interval (-) not applicable. ^1^ Results shown are interactions between sex-C-DII in linear mixed models. Models were adjusted by child’s age, child’s sex, child’s birth weight, breastfeeding type at 3 mo, socioeconomic level close to the time of birth, maternal BMI in pregnancy and mother’s education, and randomization process. % of change was calculated by the naïve method when applicable (log-transformed variables).

**Table 2:** Serum concentrations of inflammatory biomarkers at 11 years by BMI and Fat Mass.

	Body Mass Index (BMI) *					Fat Mass (kg) **		
Inflammatory Biomarkers	Normal Weight (NW) *n* = 161 *P50* (*p25*–*p75*)	Overweight (OW) *n* = 72 *P50* (*p25*–*p75*)	*p* Value * NW vs. OW	Obese (Ob) *n* = 53 *P50* (*p25*–*p75*)	*p* Value * NW vs. OB	β	95% CI	*p* Value
hsCRP (mg/L)	0.48 (0.23–1.08)	1.17 (0.50–2.42)	<0.001	3.70 (1.26–4.84)	<0.001	0.10	0.08–0.13	<0.001
IL-6 (pg/mL)	0.50 (0.22–0.87)	0.60 (0.27–0.97)	0.31	0.50 (0.19–0.91)	1.00	0.00	−0.01–0.01	0.86
Adiponectin (ng/mL)	14.55 (11.91–17.43)	14.97 (10.77–18.57)	1.00	15.01 (13.06–19.28)	0.21	0.06	−0.03–0.15	0.17
Leptin (ng/mL)	5.39 (2.67–9.78)	6.23 (2.49–12.11)	1.00	9.20 (3.78–12.89)	0.02	0.19	0.05–0.32	0.01
Adiponectin/leptin ratio	2.62 (1.28–5.66)	2.56 (1.25–4.85)	1.00	2.02 (1.08–3.97)	0.13	−0.04	−0.10–0.01	0.14

hsCRP: high sensitivity C Reactive Protein; IL-6: Interleukin 6. CI: Confidence Interval * *p* values where calculated using Dunn’s test with Bonferroni’s multiple comparison. ** Values were calculated with quantile regression.

**Table 3:** Association between C-DII and inflammatory biomarkers and hormones at 11 years.

Inflammatory Biomarkers and Hormones *	OR	95% CI	*p* Value
hsCRP			
<3 mg/L	1.00 (*Ref.*)		
≥3mg/L	1.29	0.99, 1.69	0.06
Leptin			
<1.88 ng/mL	1.00 (*Ref.*)		
1.88–<5.68 ng/mL	1.30	1.01–1.67	0.04
5.68–<13.00 ng/mL	1.38	1.0–1.78	0.01
≥13.00 ng/mL	1.34	1.02–1.76	0.03
Adiponectin			
≥19.63 ng/mL	1.00 (*Ref.*)		
<19.63–13.76 ng/mL	1.01	0.79–1.27	0.96
<13.76 ng/mL	1.01	0.80–1.28	0.92
Adiponectin/leptin ratio			
≥23.43	1.00 (*Ref.*)		
<23.43–2.45	1.52	0.97–2.39	0.07
<2.45	1.75	1.11–2.76	0.02
IL-6			
<0.55 pg/mL	1.00 (*Ref.*)		
0.55–<6.23 pg/mL	0.99	0.83–1.18	0.90
≥6.23 pg/mL	0.86	0.53–1.41	0.55

hsCRP: high sensitivity C reactive Protein; IL-6: Interleukin 6; Ref.: Reference category; CI: Confidence Interval * hsCRP was evaluated with logistic regression, while leptin, adiponectin, adiponectin/leptin ratio and IL-6 were evaluated with multivariate logistic regression. Models were adjusted by child’s sex, child’s birth weight, fat mass at 11 years, breast feeding type at 3 mo, socioeconomic level close to birth, maternal BMI at pregnancy, mother’s education and randomization process.

**Table 4:** Association between C-DII trajectories 5-11y and inflammatory biomarkers and hormones at 11 years.

Inflammatory Biomarkers and Hormones	C-DII Trajectories in Boys, *n* = 150			C-DII Trajectories in Girls, *n* = 133	
	*Trajectory 1*	*Trajectory 2*	*Trajectory 3*	*Trajectory 1*	*Trajectory 2*
**Log-hsCRP**	*Ref.*			*Ref.*	
β (95%CI)		0.01 (−0.44–0.46)	0.05 (−0.32–0.41)		0.05 (−0.41–0.51)
% change		1.05 (−35.34–57.93)	4.71 (−27.09–50.38)		5.23 (−33.44–66.53)
*p* value		0.96	0.80		0.82
**Log-IL-6**	*Ref.*			*Ref.*	
β (95%CI)		−0.028 (−0.53–0.47)	−0.008 (−0.42–0.40)		−0.006 (−0.46–0.45)
% change		−2.76 (−41.15–60.64)	−0.82 (−34.10–49.18)		−0.62 (−37.21–57.27)
*p* value		0.91	0.97		0.98
**Log-Leptin**	*Ref.*			*Ref.*	
β (95%CI)		0.29 (−0.17–0.76)	0.56 (0.19–0.94)		−0.15 (−0.55–0.24)
% change		33.98 (−15.71–112.97)	75.24 (20.49–155.05)		−14.22 (−42.46–27.88)
*p* value		0.21	<0.01		0.45
**Log-Adiponectin**	*Ref.*			*Ref.*	
β (95%CI)		−0.03 (−0.21–0.15)	0.08 (−0.06–0.23)		0.08 (−0.09–0.25)
% change		−2.92 (−18.94–16.27)	8.88 (−5.90–25.98)		8.53 (−8.58–28.86)
*p* value		0.75	0.25		0.35
**Log-Adiponectin/leptin ratio**	*Ref.*			*Ref.*	
β (95%CI)		−0.32 (−0.82–0.18)	−0.48 (−0.88–−0.07)		0.23 (−0.18–0.65)
% change		−27.54 (−56.03–19.41)	−37.89 (−58.54–−6.96)		26.52 (−16.36–91.39)
*p* value		0.20	0.02		0.26

hsCRP: high sensitivity C Reactive Protein; IL-6: Interleukin 6; Ref.: Reference category; CI: Confidence Interval. % of change was calculated by the naïve method. Models were adjusted by child’s birth weight, fat mass at 11 years, breastfeeding type at 3 mo, socioeconomic level close to birth, maternal BMI at pregnancy, mother’s education and randomization process.

## Supplementary Materials

The following are available online at https://www.mdpi.com/2072-6643/12/12/3658/s1. Table S1: Comparison of baseline characteristics between the analyzed sample and sample excluded. Table S2: General characteristics of the POSGRAD study population by follow-up age. Table S3: Food parameter distribution by C-DII tertile at 5, 7 and 11 years. Table S4: Association between C-DII, adiposity and biomarker indicators adjusting by breast button development and testosterone concentrations. Table S4: Association between C-DII and inflammatory biomarkers and hormones at 11 years, adjusting by breast button development and testosterone concentrations. Figure S1: Percentage contribution of macronutrients to total energy by C-DII tertile at 5, 7 and 11 years of age. Figure S2: C-DII trajectories from 5–11 years stratified by sex.
