# Supplementary material for: Pro-Inflammatory Diet Is Associated with Adiposity during Childhood and with Adipokines and Inflammatory Markers at 11 Years in Mexican Children
Source: Nutrients. 2020 Nov 27;12(12):3658. doi: 10.3390/nu12123658 (PMC7760203; doi:10.3390/nu12123658)
Supplement: Supplementary file 1 [file nutrients-12-03658-s001.pdf]

Online Supporting material

Supplementary Table 1. Comparison of baseline characteristics between the analyzed sample and sample excluded.

	Sample for adiposity from 5 -11 y			Sample for biomarkers at 11 y		
**Characteristics**	**Children Included** *N= 726 (74.61%)*	**Children Excluded** *N= 247 (25.39%)*	***P value***	**Children Included** *N= 286 (29.39%)*	**Children Excluded** *N= 687 (70.61%)*	***P value***
**Maternal**						
Socioecomic level,%						
Low	220 (30.30)	87 (35.22)	0.11†	74 (25.87)	233 (33.92)	0.03†
Medium	254 (34.99)	69 (27.94)		98 (34.27)	225 (32.75)	
High	252 (34.71)	91 (36.84)		114 (39.86)	229 (33.33)	
Education, n (%)						
≤9 y	273 (37.60)	94 (38.37)	0.74†	92 (32.17)	275 (40.15)	0.06†
10 to 12 y	100 (13.77)	29 (11.84)		40 (13.99)	89 (12.99)	
≥13 y	353 (48.62)	122 (49.80)		154 (53.85)	321 (46.86)	
Smoking during pregnancy, n (%)						
Yes vs. No	8 (1.10)	9 (3.64)	0.02¥	3 (1.05)	14 (2.04)	0.42¥
BMI at 18-22 weeks of pregnancy (kg/m^2), mean ± S.D	26.17 ± 4.34	25.65 ± 3.87	0.07♦	26.46 ± 4.53	25.87 ± 4.09	0.06♦
Age at 18-22 weeks (years), mean ± S.D	26.70 ± 4.77	26.46 ± 4.56	0.50♦	26.71 ± 4.54	26.60 ± 4.79	0.73♦
**Children**						
Sex,%						
Males vs. females	381 (52.48)	126 (51.01)	0.69†	152 (53.15)	355 (51.67)	0.67†
Birth weight, g						
<2500g	40 (5.54)	14 (5.74)	0.61†	17 (5.96)	37 (5.43)	0.95†
2500 to 3999g	644 (89.20)	221 (90.57)		254 (89.12)	611 (89.72)	
≥4000g	38 (5.26)	9 (3.69)		14 (4.91)	33 (4.85)	
Type of breastfeeding at 3 mo of age, %						
Exclusive - Predominant Breastfeeding	176 (24.24)	27 (24.32)	0.92†	76 (26.57)	127 (23.05)	0.30†
Partial Breastfeeding	421 (57.99)	66 (59.46)		156 (54.55)	331 (60.07)	
No breastfeeding	129 (17.77)	18 (16.22)		54 (18.88)	93 (16.88)	
Treatment with DHA, %						
Yes vs. no	359 (49.45)	127 (51.42)	0.59†	149 (52.10)	338 (49.20)	0.41†

BMI, Body Mass Index; DHA, D Docosahexaenoic acid

♦ T Student test on equality of means

† Pearson Chi-squared test

¥ Fischer's exact test

Supplementary Table 2. General characteristics of the POSGRAD study population by follow-up age

Characteristics	5 years N= 697	7 years N= 589	11 years N= 372
**Mother**			
**Socioeconomic level,** n(%)			
High	241 (34.58)	205 (34.80)	149 (40.05)
Medium	244 (35.01)	203 (34.47)	133 (35.75)
Low	212 (30.42)	181 (30.73)	90 (24.19)
**Mother's educational level,** n(%)			
High school or more	404 (57.96)	342 (58.06)	242 (65.05)
Less than high school	293 (42.04)	247 (41.94)	130 (34.95)
**Mother's BMI,** n(%)			
Normal weight	301 (43.19)	248 (42.11)	161 (43.28)
Overweight	285 (40.89)	249 (42.28)	146 (39.25)
Obese	111 (15.93)	92 (15.62)	65 (17.47)
**Children**			
**Sex,** n (%)			
Female	332 (47.63)	277 (47.03)	183 (49.19)
Male	365 (52.37)	312 (52.97)	189 (50.81)
**Type of breastfeeding (3 mo),** n(%)			
Exclusive or predominant	169 (24.25)	140 (23.77)	99 (26.61)
Partial	405 (58.11)	343 (58.23)	205 (55.11)
No breastfeeding	123 (17.65)	106 (18.00)	68 (18.28)
**C-DII**			
mean ± S.D	0.56 ± 1.46	0.62 ± 1.45	0.88 ± 1.36
**BMI,** n(%)			
Normal weight	560 (81.49)	416 (70.63)	212 (56.99)
Overweight†	86 (12.34)	94 (15.96)	91 (24.46)
Obese	43 (6.17)	79 (13.41)	69 (18.55)
**Abdominal circumference (cm)**			
mean ± S.D	54.78 ± 4.94	61.30 ± 7.60	74.00 ± 10.73
**Skinfold sum (mm)**			
mean ± S.D	15.51 ± 4.83	35.32 ± 16.51	54.34 ± 23.72
**Fat mass 11y (Kg)**			
mean ± S.D	--	--	13.88 ± 6.86
**hsCRP 11y (mg/ L)**			
P50 (p25 – p75)	--	--	0.78 (0.34 – 2.29)
**IL-6 11y (pg/mL)**			
P50 (p25 – p75)	--	--	0.50 (0.22 – 0.90)
**Adiponectin 11y (ng/mL)**			
P50 (p25 – p75)	--	--	14.71 (11.84 – 17.98)
**Leptin 11y (ng/mL)**			
P50 (p25 – p75)	--	--	6.06 (2.72 – 11.11)
**Adiponectin/leptin ratio 11y**			
P50 (p25 – p75)	--	--	2.47 (1.22 – 5.21)

* BMI: Body Mass Index; C-DII: Children's Dietary Inflammatory Index; hsCRP: high sensitivity C Reactive Protein; IL-6: Interleukin 6

Supplementary Figure 1. Percentage contribution of macronutrients to total energy by C-DII tertile at 5, 7 and 11 years of age

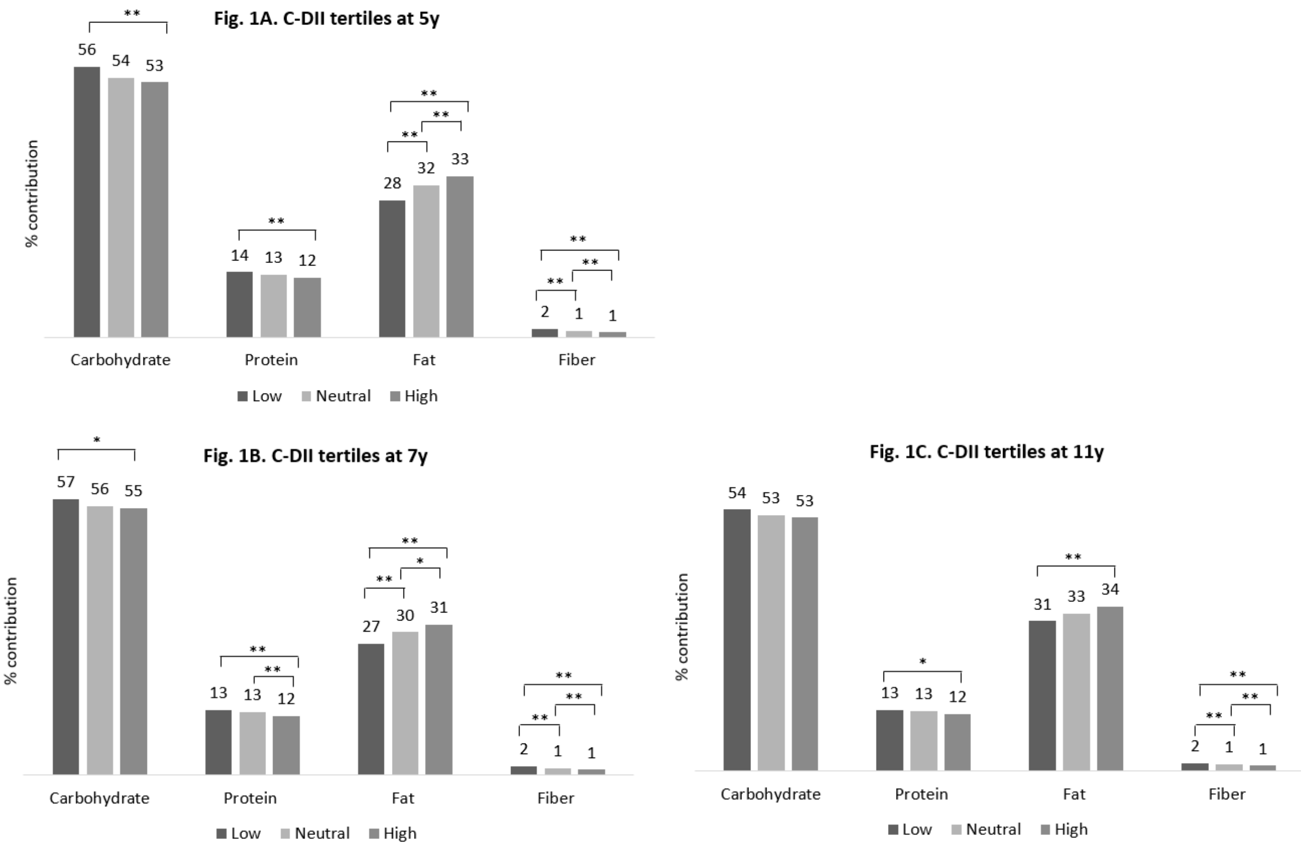

Tertiles refer to low, neutral and high inflammatory potential of the diet. ** P<0.01; * P<0.05

Supplementary Table 3. Food parameter distribution by C-DII tertile at 5, 7, and 11 years.

Food parameters	C-DII tertiles at 5y			C-DII tertiles at 7y			C-DII tertiles at 11y		
	Low<br>μ ± S.D	Neutral<br>μ ± S.D	High<br>μ ± S.D	High<br>μ ± S.D	Neutral<br>μ ± S.D	High<br>μ ± S.D	Low<br>μ ± S.D	Neutral<br>μ ± S.D	High<br>μ ± S.D
Energy (kcal)	1391.94 ± 492.55	1490.93 ± 553.51	1486.22 ± 573.78	1486.22 ± 573.78	1736.60 ± 547.56 [a]	1823.78 ± 656.90	1981.02 ± 646.61	1903.96 ± 571.03	2024.88 ± 673.65
Carbohydrates(g)	201.53 ± 76.28	206.68 ± 79.32	204.46 ± 84.89	204.46 ± 84.89	249.48 ± 81.87	258.18 ± 93.56	274.68 ± 91.95	260.32 ± 92.00	272.77 ± 113.36
Lipids (g)	45.87 ± 20.84	54.71 ± 25.41	58.06 ± 28.71 [b,c]	58.06 ± 28.71 [b,c]	59.49 ± 24.55 [a]	66.78 ± 32.16 [b,c]	71.14 ± 30.11	71.14 ± 28.01	78.57 ± 33.18
Protein (g)	48.92 ± 22.62	49.66 ± 21.19	46.83 ± 19.34	46.83 ± 19.34	58.07 ± 23.15	57.87 ± 27.07	63.40 ± 23.17	59.88 ± 18.59	60.27 ± 24.53
Fiber (g)	11.92 ± 6.84	10.28 ± 6.86 [a]	7.50 ± 4.69 [b,c]	7.50 ± 4.69 [b,c]	11.39 ± 5.27 [a]	9.44 ± 4.24 [b,c]	16.42 ± 7.18	13.17 ± 5.20 [a]	10.81 ± 5.27 [b,c]
Alcohol (g)	0.06 ± 0.42	0.11 ± 0.81	0.04 ± 0.24	0.04 ± 0.24	0.04 ± 0.19	0.09 ± 0.75	0.07 ± 0.51	0.10 ± 0.33	0.09 ± 0.38
Cholesterol (mg)	221.72 ± 177.02	224.69 ± 150.64	220.35 ± 136.28	220.35 ± 136.28	258.74 ± 177.46	269.12 ± 176.51 [b]	278.90 ± 207.37 [d]	272.74 ± 205.08	305.29 ± 205.81
SFA (g)	18.98 ± 8.35	23.97 ± 11.34 [a]	25.40 ± 12.26 [b]	25.40 ± 12.26 [b]	23.55 ± 10.71 [a]	27.37 ± 13.41 [b,c]	25.84 ± 12.78	26.66 ± 11.49	32.59 ± 17.02 [b,c]
MUFA (g)	16.48 ± 8.56	18.70 ± 9.01 [a]	19.85 ± 9.71 [b]	19.85 ± 9.71 [b]	19.32 ± 8.63	21.63 ± 10.78 [b,c]	23.17 ± 10.19	24.19 ± 10.74 [a]	26.41 ± 12.25
PUFA (g)	7.82 ± 4.89	8.41 ± 5.33	8.15 ± 6.01	8.15 ± 6.01	10.67 ± 6.76	10.56 ± 7.07	15.76 ± 9.36	14.87 ± 8.11	14.61 ± 8.18
Vitamin A (RAE)	732.23 ± 355.00	577.59 ± 303.77 [a]	425.52 ± 263.50 [b,c]	425.52 ± 263.50 [b,c]	542.51 ± 292.61 [a]	438.58 ± 292.75 [b,c]	846.77 ± 515.94	474.69 ± 308.02 [a]	408.57 ± 265.02 [b]
β-carotene (μg)	1500.98 ± 2149.09	625.45 ± 1305.68 [a]	293.44 ± 383.30 [b,c]	293.44 ± 383.30 [b,c]	750.36 ± 1021.10 [a]	381.32 ± 388.53 [b,c]	1890.07 ± 2295.02	979.64 ± 1756.23 [a]	395.67 ± 485.58 [b,c]
Vitamin C (mg)	160.48 ± 118.	106.74 ± 85.50 [a]	60.73 ± 60.83 [b,c]	60.73 ± 60.83 [b,c]	109.98 ± 86.79 [a]	64.72 ± 69.56 [b,c]	169.81 ± 118.42	82.66 ± 67.92 [a]	50.27 ± 56.80 [b,c]
Vitamin D (IU)	78.48 ± 70.43	69.59 ± 68.70	56.34 ± 51.72 [b,c]	56.34 ± 51.72 [b,c]	79.40 ± 90.57 [a]	68.93 ± 89.84 [b]	170.42 ± 133.60	147.60 ± 117.00	142.30 ± 138.46
Vitamin E (mg)	5.07 ± 2.92	3.95 ± 2.60 [a]	3.03 ± 2.14 [b,c]	3.03 ± 2.14 [b,c]	4.20 ± 2.68 [a]	3.21 ± 1.97 [b,c]	7.44 ± 4.23	5.24 ± 3.15 [a]	4.60 ± 2.88 [b]
Thiamin (mg)	1.12 ± 0.50	1.05 ± 0.47	0.88 ± 0.46 [b,c]	0.88 ± 0.46 [b,c]	1.19 ± 0.49 [a]	1.04 ± 0.49 [b,c]	1.58 ± 0.74	1.20 ± 0.49 [a]	1.18 ± 0.57 [b]
Riboflavin (mg)	1.71 ± 0.74	1.57 ± 0.68 [a]	1.26 ± 0.60 [b,c]	1.26 ± 0.60 [b,c]	1.55 ± 0.69 [a]	1.38 ± 0.76 [b,c]	2.03 ± 1.04	1.52 ± 0.76 [a]	1.44 ± 0.77 [b]
Niacin (g)	14.14 ± 7.88	11.53 ± 6.15 [a]	9.32 ± 5.54 [b,c]	9.32 ± 5.54 [b,c]	13.27 ± 6.19 [a]	11.11 ± 7.39 [b,c]	17.91 ± 8.00	12.68 ± 5.28 [a]	11.58 ± 5.83 [b]
B6 (mg)	1.31 ± 0.80	1.03 ± 0.59 [a]	0.77 ± 0.49 [b,c]	0.77 ± 0.49 [b,c]	1.15 ± 0.61 [a]	0.86 ± 0.54 [b,c]	1.57 ± 0.97	1.06 ± 0.53 [a]	0.83 ± 0.47 [b,c]
Folate (DFE)	488.59 ± 314.55	384.79 ± 248.64 [a]	262.73 ± 185.66 [b,c]	262.73 ± 185.66 [b,c]	338.54 ± 193.77 [a]	262.20 ± 192.67 [b,c]	570.99 ± 427.20	391.42 ± 211.70 [a]	408.42 ± 352.63 [b]
B12 (μg)	2.56 ± 2.50	2.83 ± 2.56	2.64 ± 3.05	2.64 ± 3.05	2.76 ± 2.47	2.47 ± 2.15	3.39 ± 2.77	2.97 ± 2.80	2.44 ± 1.75 [b]
Iron (mg)	14.30 ± 6.60	12.72 ± 5.89 [a]	10.33 ± 5.18 [b,c]	10.33 ± 5.18 [b,c]	13.73 ± 5.74 [a]	12.68 ± 5.81 [b]	17.52 ± 8.09	13.82 ± 5.44 [a]	13.28 ± 6.04 [b]
Magnesium (mg)	237.39 ± 92.04	218.10 ± 92.90 [a]	170.75 ± 76.98 [b,c]	170.75 ± 76.98 [b,c]	297.29 ± 111.37	260.15 ± 112.92 [b,c]	341.94 ± 123.35	300.85 ± 106.16 [a]	251.49 ± 102.34 [b,c]
Selenium (μg)	59.10 ± 33.94	61.68 ± 29.55	59.75 ± 28.74	59.75 ± 28.74	75.05 ± 35.17	74.75 ± 35.61	76.72 ± 33.79	73.25 ± 27.36	71.68 ± 33.43
Zinc (mg)	9.52 ± 4.10	8.97 ± 4.34	7.43 ± 3.89 [b,c]	7.43 ± 3.89 [b,c]	8.94 ± 3.96 [a]	8.02 ± 3.94 [b,c]	10.49 ± 4.50	8.54 ± 3.41 [a]	8.14 ± 4.18 [b]

*Tertiles refer to low, neutral and high inflammatory potential of diet

[a]Low vs. Neutral $p<0.01$, [b]Low vs. High $p<0.01$, [c]Neutral vs. High $p<0.01$

Supplementary Figure 2. C-DII trajectories from 5-11 years stratified by sex

Supplementary Fig. 2a. C-DII trajectories in boys, 5-11y

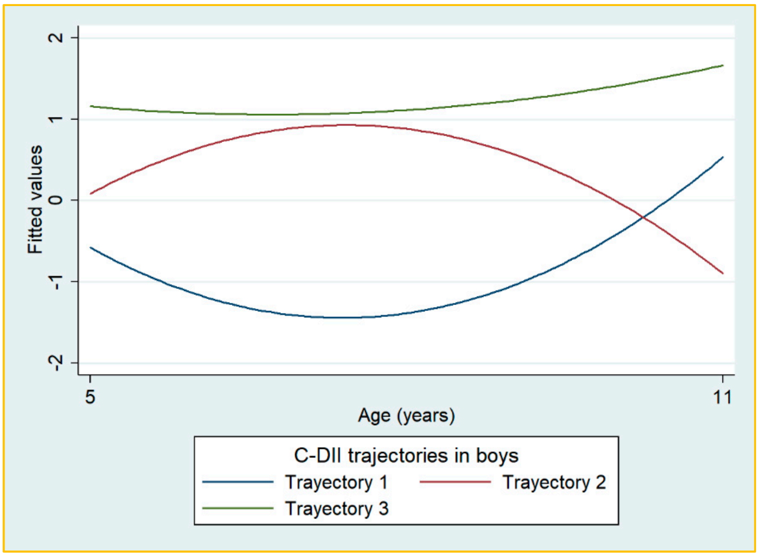

Supplementary Fig. 2b. C-DII trajectories in girls, 5-11y

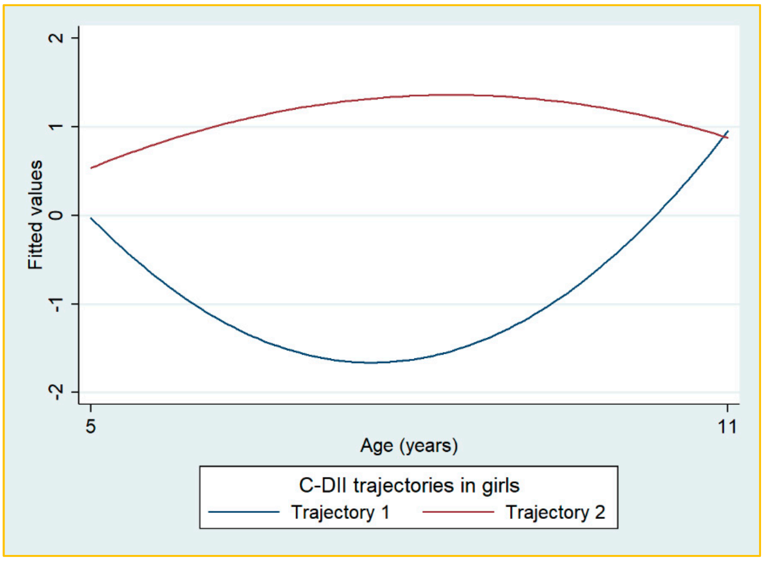

Supplementary Table 4. Association between C-DII, adiposity, and biomarker indicators adjusting by breast button development and testosterone concentrations

Children's Dietary Inflammatory Index 5-11y N= 311	BMI Z score	Log-Abdominal circumference	Log-Skinfold sum
**C-DII Males**			
β (95% CI)	-0.011 (-0.058 – 0.036)	-0.003 (-0.007 – 0.002)	-0.005 (-0.022 – 0.011)
% of change	-	-0.30 (-0.70 – 0.20)	-0.50 (-2.18 – 1.11)
P value	0.65	0.25	0.52
**C-DII Females**	-		
β (95% CI)	0.079 (0.027 – 0.131)	0.007 (0.002 – 0.012)	0.028 (0.010 – 0.047)
% of change	-	0.70 (0.20 – 1.21)	2.84 (1.01 – 4.81)
P value	<0.01	<0.01	<0.01
**P for C-DII - sex interaction**	0.01	<0.01	<0.01

BMI: Body Mass Index; CI: Confidence Interval ;(-) not applicable. [1] Results shown are interactions between sex-C-DII in linear mixed models. Models were adjusted by child's age, child's sex, child's birth weight, breastfeeding type at 3 mo, socioeconomic level close to the time of birth, maternal BMI in pregnancy and mother's education, breast button development, testosterone concentrations, and randomization process. % of change was calculated by the naïve method when applicable (log-transformed variables).

Supplementary Table 5. Association between C-DII and inflammatory biomarkers and hormones at 11 years, adjusting by breast button development and testosterone concentrations

Inflammatory biomarkers and hormones at 11y*	OR**	95% CI	P value
hsCRP			
<3 mg/L	1.00 (*ref.*)		
≥3mg/L	1.29	0.99, 1.69	0.06
Leptin (n= 207)			
<1.88 ng/mL	1.00 (*ref.*)		
1.88 - <5.68 ng/mL	1.23	0.91 – 1.67	0.18
5.68 - <13.00 ng/mL	1.48	1.08 – 2.03	0.01
≥13.00 ng/mL	1.36	0.95 – 1.93	0.09
Adiponectin (n= 207)			
≥19.63 ng/mL	1.00 (*ref.*)		
<19.63 – 13.76 ng/mL	0.96	0.70 – 1.33	0.81
<13.76 ng/mL	1.08	0.78 – 1.48	0.64
Adiponectin/leptin ratio (n= 207)			
≥23.43	1.00 (*ref.*)		
<23.43 – 2.45	1.52	0.97 – 2.39	0.07
<2.45	1.75	1.10 – 2.76	0.02
IL-6			
<0.55 pg/mL	1.00 (*ref.*)		
0.55 - <6.23 pg/mL	0.99	0.83 – 1.18	0.90
≥6.23 pg/mL	0.86	0.53 – 1.41	0.55

hsCRP: high sensitivity C reactive Protein; IL-6: Interleukin 6. Ref.: Reference category; CI: Confidence Interval. *hsCRP was evaluated with logistic regression, while leptin, adiponectin, adiponectin/leptin ratio and IL-6 were evaluated with multivariate logistic regression. Models were adjusted by child's sex, child's birth weight, fat mass at 11 years, breast feeding type at 3 mo, socioeconomic level close to birth, maternal BMI at pregnancy, mother's education, breast button development, testosterone concentrations, and randomization process.
